# Correction: Charge-voltage curves of Shaker potassium channel are not hysteretic at steady state

**DOI:** 10.1085/jgp.20211288302132023C

**Published:** 2023-02-27

**Authors:** John Cowgill, Baron Chanda

Vol. 155, No. 3 | https://doi.org/10.1085/jgp.202112883 | January 24, 2023

The authors regret that in the original version of Fig. S2 A, an incorrect constant was given for the Os to Cu transition. The correct rate is 0.0500625, as seen in the corrected figure here. The actual simulations were done with the correct rate constants; to assuage any concerns, the authors have also rerun the simulations to confirm that the rest of the figures are unchanged.

**Figure S2.**
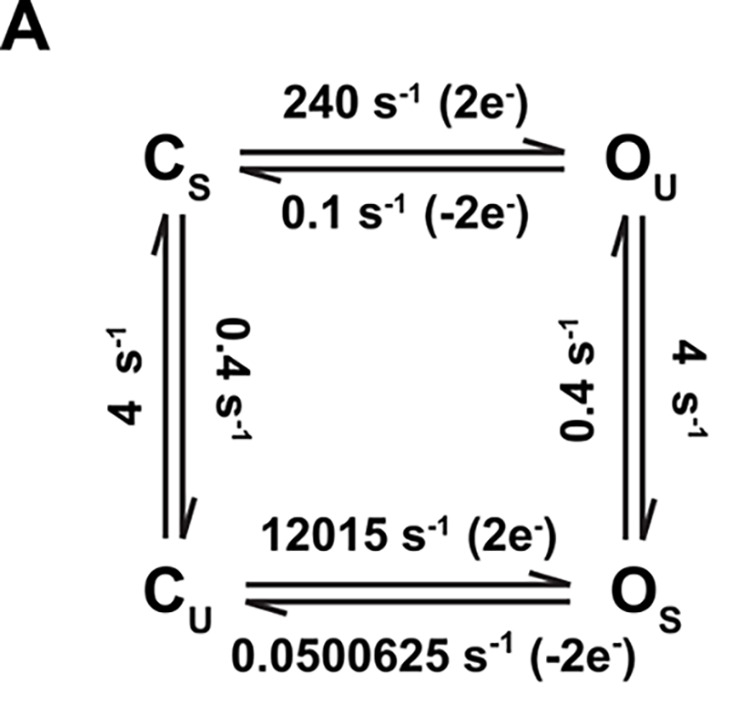
**Mode shift does not generate hysteresis in Q-V curve at equilibrium.** **(A)** Four-state kinetic model representing a classical mode-shifted gating scheme used for simulating data in B and C according to similar protocol as in Fig. 2 A.

In addition, the authors added two sentences to the end of the Fig. 2 legend for clarity. The added text is indicated in bold underline.

**Figure 2.** **Hysteresis in Shaker gating decreases with increasing pulse duration.** **(A)** Capacitive- and leak-subtracted gating current recordings using 50-ms test pulses from polarized holding potentials (left) or depolarized holding potentials (right). Both recordings are from the same oocyte and shown on the same scale for direct comparison. The off-gating pulse was used for integration of gating charge and was set to 0 mV due to fast gating current kinetics and minimal ionic current contamination. In the polarized holding potential condition, a 200-ms prepulse to −110 mV from a holding potential of −80 mV was used to minimize the time the oocyte was held at extreme potentials. **(B)** Q-V relationships for Shaker W434F recorded with variable pulse durations in depolarizing (black) or hyperpolarizing (red) direction. The shift reported in each graph represents the difference in the median voltage extracted from the polarized and depolarized conditions. Error bars show standard error for at least three oocytes for each condition. **Note that the y axis represents 1 − Q/Qmax since the integrated charge at the reference potential will be maximal for test pulses to negative potentials. However, we maintain the Q/Qmax label for consistency with the convention of the field.**

The errors appear in PDFs downloaded before February 15, 2023.

